# [^11^C]PK11195-PET Brain Imaging of the Mitochondrial Translocator Protein in Mitochondrial Disease

**DOI:** 10.1212/WNL.0000000000012033

**Published:** 2021-06-01

**Authors:** Jelle van den Ameele, Young T. Hong, Roido Manavaki, Antonina Kouli, Heather Biggs, Zoe MacIntyre, Rita Horvath, Patrick Yu-Wai-Man, Evan Reid, Caroline H. Williams-Gray, Ed T. Bullmore, Franklin I. Aigbirhio, Tim D. Fryer, Patrick F. Chinnery

**Affiliations:** From the Departments of Clinical Neurosciences (J.v.d.A., Y.T.H., A.K., H.B., Z.M., R.H., P.Y.-W.M., C.H.W.-G., F.I.A., T.D.F., P.F.C.), Radiology (R.M.), Medical Genetics (E.R.), and Psychiatry (E.T.B.), Cambridge Institute for Medical Research (E.R.), Cambridge Biomedical Campus, and MRC Mitochondrial Biology Unit (J.v.d.A., P.F.C.), University of Cambridge; Moorfields Eye Hospital NHS Foundation Trust (P.Y.-W.M.); and Institute of Ophthalmology (P.Y.-W.M.), University College London, UK.

## Abstract

**Objective:**

To explore the possibilities of radioligands against the mitochondrial outer membrane translocator protein (TSPO) as biomarkers for mitochondrial disease, we performed brain PET-MRI with [^11^C]PK11195 in 14 patients with genetically confirmed mitochondrial disease and 33 matched controls.

**Methods:**

Case–control study of brain PET-MRI with the TSPO radioligand [^11^C]PK11195.

**Results:**

Forty-six percent of symptomatic patients had volumes of abnormal radiotracer binding greater than the 95th percentile in controls. [^11^C]PK11195 binding was generally greater in gray matter and significantly decreased in white matter. This was most striking in patients with nuclear *TYMP* or mitochondrial m.3243A>G *MT-TL*1 mutations, in keeping with differences in mitochondrial density seen postmortem. Some regional binding patterns corresponded to clinical presentation and underlying mutation, even in the absence of structural changes on MRI. This was most obvious for the cerebellum, where patients with ataxia had decreased binding in the cerebellar cortex, but not necessarily volume loss. Overall, there was a positive correlation between aberrant [^11^C]PK11195 binding and clinical severity.

**Conclusion:**

These findings endorse the use of PET imaging with TSPO radioligands as a noninvasive in vivo biomarker of mitochondrial pathology.

**Classification of Evidence:**

This study provides Class III evidence that brain PET-MRI with TSPO radioligands identifies mitochondrial pathology.

Mitochondrial diseases have emerged as among the most common inherited neurologic disorders and together affect about 1 in 5,000 of the UK population.^1^ Mitochondrial diseases are progressive multisystem disorders that can present at any stage in life and often affect the CNS.^2^ Curative treatments are lacking and management is largely based on symptomatic therapies and maximizing quality of life.^3-5^ Although there are a growing number of agents being tested in preclinical animal models, few studies have demonstrated efficacy in humans.^6-8^ This reflects in part the lack of objective clinical biomarkers that show change over a practical timescale or allow subgroup characterization, essential prerequisites to provide evidence of efficacy in a heterogeneous rare disease cohort.^9,10^

The isoquinoline PK11195 selectively binds to the 18 kDa translocator protein (TSPO),^11,12^ thought to be involved in cholesterol transport across the outer mitochondrial membrane.^13-15^ [^11^C]PK11195 PET imaging of TSPO has mainly been used as a marker of neuroinflammation and microglial activation, based on strong radiotracer accumulation in ischemic and inflammatory brain lesions.^16-18^ However, it is increasingly appreciated that TSPO ligand binding as a measurement of microglial activation is an oversimplification, and altered TSPO abundance is likely affected by many other disease-specific processes.^19,20^ Given its localization to mitochondria,^13-15^ we sought to explore the utility of [^11^C]PK11195 PET imaging of TSPO as an in vivo biomarker of mitochondrial pathology in the brain, with a view to monitoring progression of mitochondrial disease.

## Methods

### Classification of Evidence

The primary research objective was to explore possibilities of TSPO radioligands as in vivo noninvasive biomarkers for mitochondrial disease, based on the localization of TSPO to the outer mitochondrial membrane in all cell types of the brain. This study provides Class III evidence that brain PET-MRI with TSPO radioligands identifies mitochondrial pathology.

### Standard Protocol Approvals, Registrations, and Patient Consents

Patients with genetically confirmed mitochondrial disease were recruited through a specialist mitochondrial disease clinic at Addenbrooke's Hospital, Cambridge University Hospitals NHS Foundation Trust, United Kingdom. Fifteen patients consented to undergo PET-MRI. The scan of patient 13 was cancelled because of an upper respiratory tract infection and this patient was excluded from the analysis. Human phenotype ontology terms for each patient were assigned based on electronic health records. Modified Rankin Scale (mRS) score and Scale for the Assessment and Rating of Ataxia (SARA) were calculated based on available clinical examinations in the electronic health records. The research protocol was approved by a National Research Ethics Service committee (REC ID: 16/LO/2093). The healthy control cohort comprised subjects from projects in depression (n = 25; REC ID: 15/EE/0092) and Parkinson disease (n = 8; REC ID: 16/EE/0445; recruited via the NIHR Cambridge Bioresource) that used the same data acquisition protocol on the same PET-MRI scanner. All these projects received approval from the Administration of Radioactive Substances Advisory Committee and all participants provided written informed consent in accordance with the Declaration of Helsinki.

### PET Data Acquisition

All participants underwent PET and MRI in a single session on a GE Signa PET-MR scanner (GE Healthcare), which can simultaneously acquire PET and 3T MRI data. [^11^C]PK11195 was injected over approximately 30 seconds and list-mode PET data were acquired for 75 minutes. The median (interquartile range [IQR]) injected activity was 397 (62) MBq with corresponding injected mass values of 3.8 (3.8) μg. Injected activity per unit body weight (MBq/kg) and injected mass were not significantly different between the mitochondrial disease and control groups. Attenuation correction included the use of a multisubject atlas method^21^ and improvements to the MRI brain coil component.^22^ Other data corrections (dead time, randoms, normalization, scatter, sensitivity, and decay) were as implemented on the scanner. Dynamic sinograms were reconstructed into 128 × 128 × 89 arrays (2.0 × 2.0 × 2.8 mm voxel size) using time-of-flight ordered subsets expectation maximization,^23^ with 6 iterations, 16 subsets, and no smoothing. Brain T1 and T2 MRI acquired as part of the protocol were reviewed as part of routine clinical radiology reporting.

### Image Processing and Kinetic Analysis

Each emission image series was aligned using SPM12 (fil.ion.ucl.ac.uk/spm/software/spm12/) to ameliorate the effect of motion, then rigidly registered to the high-resolution (isotropic 1.0 mm), volumetric T1-weighted MRI acquired during PET data acquisition. Using a version of the Hammersmith atlas (brain-development.org) defined on the ICBM 152 2009a T1 template with modified posterior fossa regions, 33 regions of interest (ROIs) including aggregated regions for frontal, parietal, occipital, and temporal lobes; cingulate; and cerebellum were delineated on the T1-weighted MRI of each participant using the inverse of spatial normalization parameters determined with SyN^24^ implemented in the Advanced Normalisation Tools software package (picsl.upenn.edu/software/ants/). Regional time-activity curves were extracted following the application of geometric transfer matrix (GTM) partial volume correction (PVC)^25^ to each of the dynamic PET images. The GTM step was performed twice, with the ROIs in each case multiplied by a gray or white matter binary mask (>50% on the SPM12 probability map smoothed to PET spatial resolution). Multiple additional regions were defined to provide full spatial coverage for GTM PVC.

To quantify [^11^C]PK11195 binding, nondisplaceable binding potential (BP_ND_) was determined, both regionally and at the voxel level, using a basis function implementation of a simplified reference tissue model that includes correction for vascular binding,^26^ with the reference tissue defined using supervised cluster analysis.^27^ For the gray and white matter masked ROIs, the reference tissue used corresponded to the gray matter and white matter kinetic class, respectively. To provide a global BP_ND_ metric for both gray matter and white matter, the volume-weighted average of the BP_ND_ values in the corresponding ROIs was determined. Similarly, 2 BP_ND_ maps were produced per subject using either the gray matter or white matter reference tissue input. Prior to parametric mapping, the dynamic PET images were smoothed with a 4 mm full width at half maximum (FWHM) Gaussian. To facilitate voxel-wise statistical analysis, BP_ND_ maps were normalized to ICBM 152 2009a template space using the parameters obtained with SyN spatial normalization of the coregistered T1-weighted MRI. All images are shown in radiologic format (i.e., the left of the brain is on the right of the image).

### Statistical Analysis

#### Demographics

The age and sex distributions of the control and mitochondrial disease groups were compared using the Mann-Whitney *U* and Fisher exact tests, respectively.

#### Regional [^11^C]PK11195 Binding Potential

Given that tissue class masking resulted in the loss of certain ROIs for some or all subjects, and resulted in very small volumes for other ROIs, the number of regions reported for gray and white matter is 17 and 12, respectively, with bilateral regions combined. For each region, linear regression with group, age, and sex as factors was applied to the regional BP_ND_ values to determine the group effect (jamovi version 0.9.6.9; jamovi.org). The *p* values reported as significant are those that survive correction for family-wise error rate across ROIs using the Holm procedure. The same linear regression approach was applied to the global BP_ND_ values. Graphs were generated in R (The R Foundation; r-project.org). Box-and-whisker plots depict median, IQR (box), and 1.5 IQR below and above the first and third quartiles, respectively (whiskers); data points indicate the value of individual patients. In violin plots, data points indicate values within ROIs.

#### Voxel-wise [^11^C]PK11195 Binding Potential

Prior to statistical testing with SPM12, BP_ND_ maps were multiplied by a brain mask and smoothed with an 8-mm FWHM Gaussian. For each mitochondrial disease participant, each of the 2 BP_ND_ maps was compared to the corresponding BP_ND_ maps from the control group using the 2-sample *t* test. This process was repeated for each control participant against the remaining controls. The resultant *T* statistic maps were converted to *p* value maps and masked with subject-specific gray matter and white matter binary masks to determine the number of voxels with *p* < 0.001 (uncorrected) within each region for each tissue class. Voxels with *p* < 0.001 were summed across all ROIs and divided by the subject-specific total number of voxels in the ROIs to produce a global voxel fraction, which was compared between controls and patients with mitochondrial disease using linear regression with group, age, and sex as factors, as for the regional BP_ND_ values. In addition, for each patient with mitochondrial disease, the global voxel fraction was compared to the 95th percentile of the control global voxel fraction distribution, with the 95th percentile determined using bootstrapping (100,000 samples) in MATLAB R2019b (Mathworks Inc.). For display purposes, the *T*-statistic maps were converted to −log_10_(*p*) maps and the 2 maps were combined into a single map using binary gray matter and white matter masks to select voxels from the corresponding tissue class. Correlations of global voxel fractions with clinical severity scales were calculated as Pearson correlation coefficient in R.

### Data Availability

The raw data that support the findings of this study are available on request from the corresponding author. All patient −log10(*p*) maps are displayed in appendix e-1 (available from Dryad, doi.org/10.5061/dryad.zs7h44j7s) as overlay of −log10(*p*) on the ICBM 152 2009a T1 MRI template for voxels with significantly increased (top) or decreased (bottom) binding potential (*p* < 0.05; *p* ≤ 0.001 in red) for each patient compared to controls. The scale shown for patient 1 applies for all other images.

## Results

### Patient Characteristics

Fourteen patients with genetically confirmed mitochondrial disease underwent PET-MRI. Demographics, clinical characteristics, mutations, and MRI findings of all patients are described in the table. mtDNA haplotypes were not available. Patient 6 is an asymptomatic family member of patient 5, with low heteroplasmy levels of the *MT-ND6* m.14487T>C mutation (as previously described in reference 28) and was excluded from statistical analyses of affected individuals. The control dataset included 33 healthy volunteers whose age and sex distributions matched those of the patient group (*p* = 0.826 for age; *p* = 0.185 for sex; figure e-1 available from Dryad, doi.org/10.5061/dryad.zs7h44j7s).

**Table:**
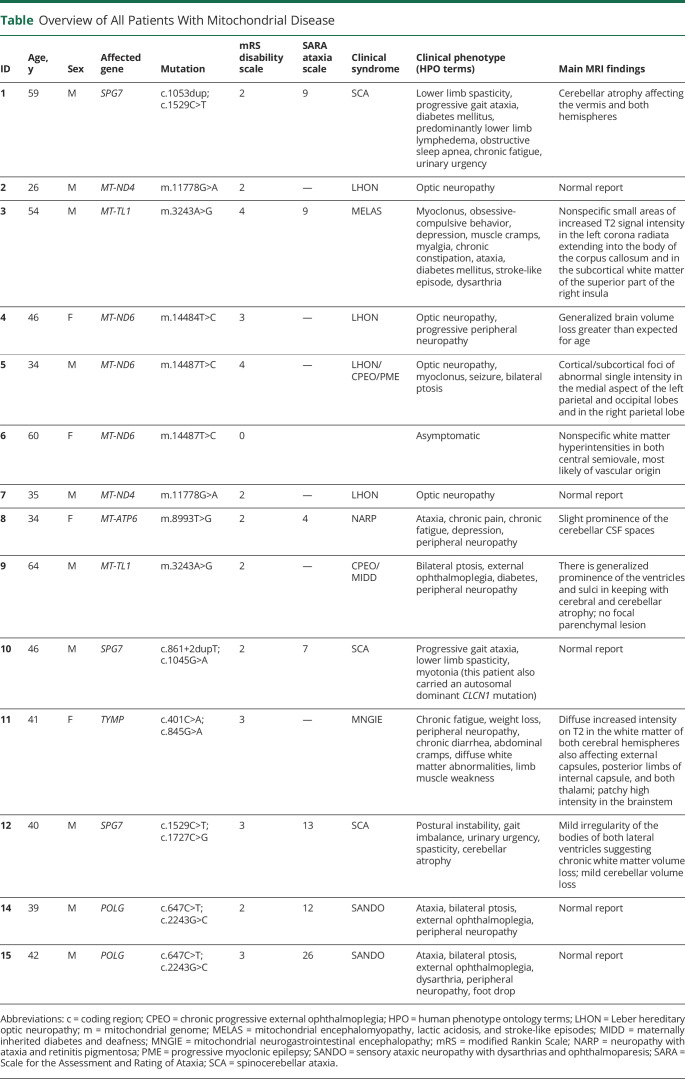
Overview of All Patients With Mitochondrial Disease

### Mitochondrial Disease Affects [^11^C]PK11195 Binding in the Brain

**Figure 1:**
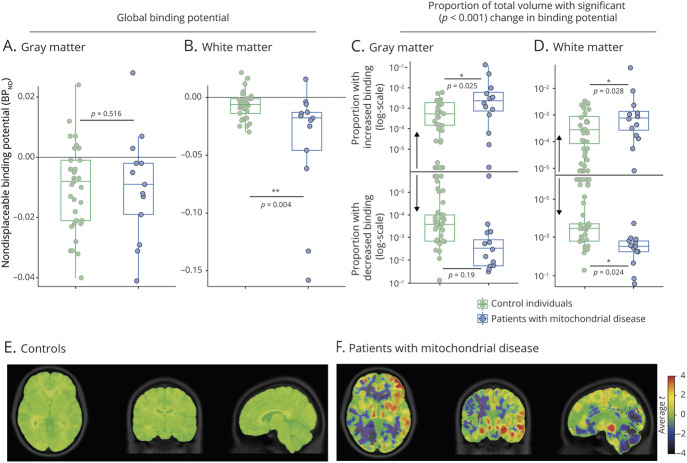
Mitochondrial Disease Affects [^11^C]PK11195 Binding Potential (A, B) Global [^11^C]PK11195 nondisplaceable binding potential (BP_ND_) in (A) gray matter or (B) white matter for each control (green) and patient with mitochondrial disease (blue). (C, D) Fraction of voxels in (C) gray matter or (D) white matter for each individual from the control (green) or mitochondrial disease group (blue) where BP_ND_ was significantly different (*p* < 0.001) from the control population at the voxel level. Y-axis is log-transformed. *p* Values for A–D are calculated by age- and sex-corrected linear regression. (E, F) Average of individual *T* statistic maps for (E) controls and (F) patients with mitochondrial disease compared to the control cohort, overlaid on the ICBM 152 2009a T1 MRI template.

We compared radiotracer binding characteristics in the gray and white matter across the brain between patients and controls. In gray matter, global BP_ND_ was comparable between both groups (*p* = 0.516), with the values for most patients falling within the control range (figure 1A). In contrast, across white matter, global BP_ND_ was lower in patients with mitochondrial disease than in controls (*p* = 0.004), mainly determined by a large decrease in BP_ND_ in 2 or 3 of the patients (figure 1B; see below).

Across patients and different regions of the brain, the volume of significantly increased or reduced BP_ND_ was highly heterogeneous, but overall, the proportion of gray or white matter with significantly (*p* < 0.001) increased or reduced BP_ND_ was significantly higher in patients than in controls (gray matter: *p* = 0.025 for voxels with increased BP_ND_; white matter: *p* = 0.028 for increased and *p* = 0.024 for decreased BP_ND_) (figure 1, C–F; figure e-2 available from Dryad, doi.org/10.5061/dryad.zs7h44j7s). For 6/13 (46%) symptomatic patients, the proportion of voxels with significantly increased or decreased BP_ND_ was higher than the 95th percentile of controls. Patient 6, an asymptomatic carrier, had the lowest volume of abnormal binding (0.1 mL). Together, these findings indicate that, although mitochondrial disease does not cause a homogeneous and reproducible change in BP_ND_, it can significantly affect [^11^C]PK11195 binding in large parts of the brain in individual patients.

### Regional [^11^C]PK11195 Binding Characteristics

**Figure 2:**
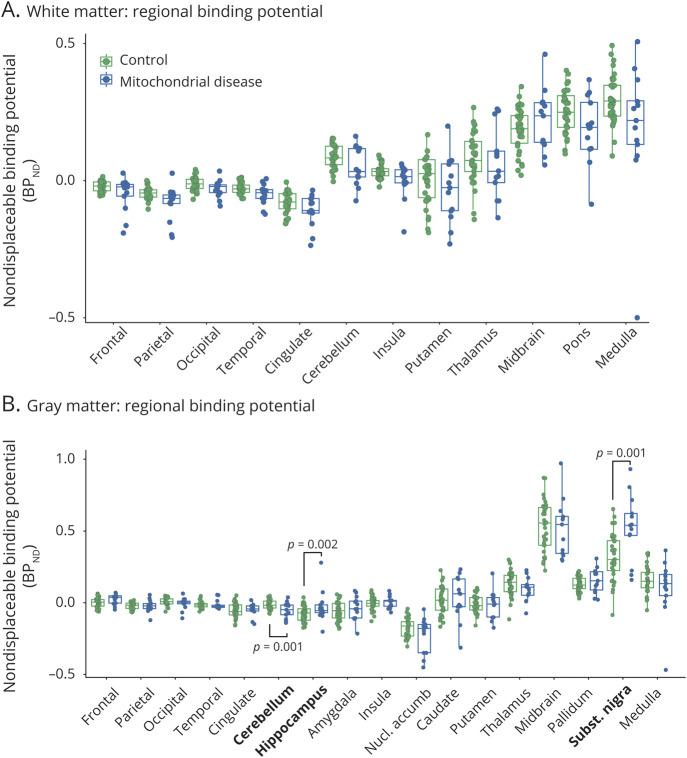
Regional [^11^C]PK11195 Binding Potential in Gray and White Matter (A, B) [^11^C]PK11195 nondisplaceable binding potential (BP_ND_) in (A) white matter or (B) gray matter of each indicated region of interest (ROI) for controls (green) or patients with mitochondrial disease (blue). ROIs with significant change following correction for multiple comparisons are indicated in bold. *p* Values are calculated by age- and sex-corrected linear regression.

To assess whether [^11^C]PK11195 binding in specific brain regions would be preferentially affected by mitochondrial dysfunction, BP_ND_ was determined in 12 white matter (figure 2A) and 17 gray matter ROIs (figure 2B). Consistent with the decrease in global BP_ND_ in white matter (figure 1B), 11/12 white matter ROIs (not midbrain; figure 2A) showed decreased radiotracer binding in patients with mitochondrial disease. However, when corrected for multiple comparisons, these region-specific white matter differences did not reach significance. In contrast, in gray matter, following correction for multiple comparisons, regional BP_ND_ was significantly lower in the cerebellar gray matter of patients with mitochondrial disease compared to controls (*p* = 0.001), while BP_ND_ in the hippocampus (*p* = 0.002) and substantia nigra (*p* = 0.001) were significantly increased (figure 2B).

### Decreased [^11^C]PK11195 BP in the Cerebellum of Patients With Ataxia

**Figure 3:**
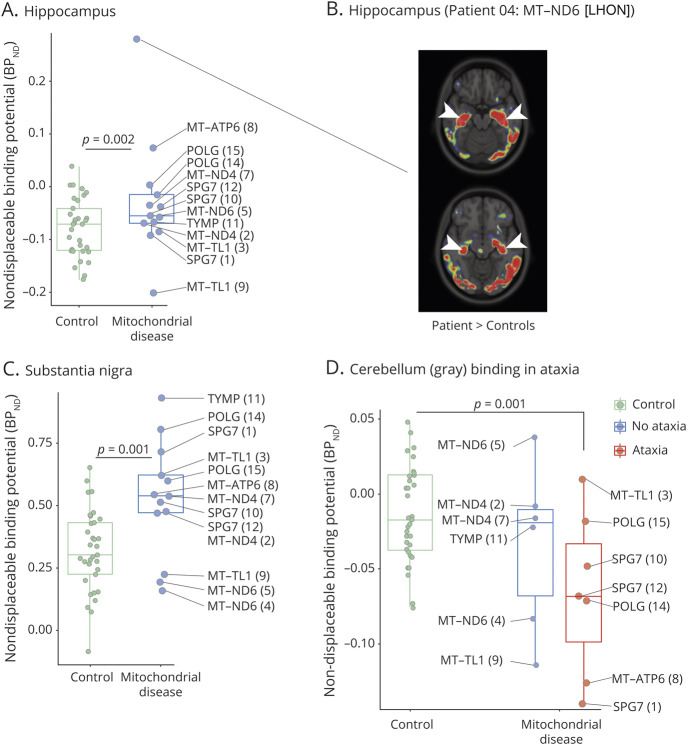
Increased and Decreased [^11^C]PK11195 Binding Potential in Gray Matter Regions (A–C) [^11^C]PK11195 nondisplaceable binding potential (BP_ND_) in (A) hippocampus or (C) substantia nigra (as shown in figure 2B) for controls (green) or patients with mitochondrial disease (blue). Mutated gene and patient number in brackets associated with each datapoint are indicated. (B) Overlay of −log_10_(*p* value) of voxels with significantly increased (patient > controls) BP_ND_ (*p* < 0.05; *p* ≤ 0.001 in red) on the ICBM 152 2009a T1 MRI template for patient 4, the highest datapoint in (A). The arrowheads indicate the hippocampus. (D) [^11^C]PK11195 BP_ND_ in cerebellar gray matter for controls (green) and patients with mitochondrial disease without (blue) or with (red) ataxia. *p* Values are calculated by age- and sex-corrected linear regression. LHON = Leber hereditary optic neuropathy.

We examined whether the significant differences observed in gray matter of the cerebellum, hippocampus, and substantia nigra were due to specific mutations or clinical characteristics (figure 3). The most striking outlier was patient 4 with Leber hereditary optic neuropathy (LHON) and peripheral neuropathy due to a *MT-ND6* m.14484T>C mutation, where we observed a strong increase in binding in both hippocampi (Figure 3, A and B). This patient had generalized brain atrophy as reported on MRI, but no clinical signs or symptoms suggestive of hippocampal dysfunction (table). Increased BP_ND_ values in the substantia nigra (figure 3C) also did not correlate with the presence of parkinsonism or other extrapyramidal symptoms (table), despite these symptoms being part of the clinical spectrum in mitochondrial disease.^29^

Interestingly, the 2 patients with the lowest regional BP_ND_ in cerebellar gray matter both presented with ataxia (patient 1 with an *SPG7* mutation and patient 8 with an *MT-ATP6* mutation) (figure 3D). Subgroup analysis of all patients with ataxia (7/13) showed a significant decrease in regional BP_ND_ (*p* = 0.001) compared to controls (figure 3D). This was not always accompanied by atrophy on brain MRI (table), suggesting that clinically relevant changes in [^11^C]PK11195 binding patterns may precede or occur independently of gross structural abnormalities.

### Regional Differences in Decreased [^11^C]PK11195 Binding in White Matter Caused by *MT-TL1* and *TYMP* Mutations

**Figure 4:**
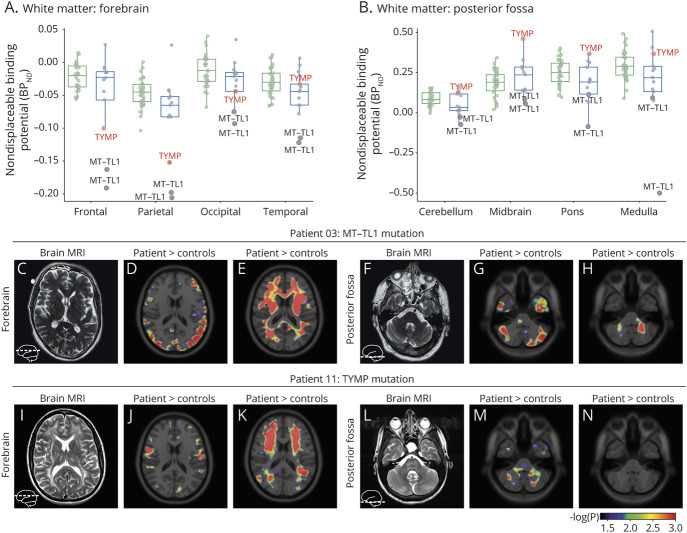
*MT-TL1* and *TYMP* Mutations Cause Decreased White Matter [^11^C]PK11195 Binding (A, B) [^11^C]PK11195 nondisplaceable binding potential (BP_ND_) in the indicated white matter regions of interest of the (A) forebrain and (B) posterior fossa for controls (green) or patients with mitochondrial disease (blue). Datapoints from patients 3, 9 (*MT-TL1* mutations, gray), and 11 (*TYMP* mutations, red) are highlighted. (C–N) Overlay on the ICBM 152 2009a T1 MRI template of −log_10_(*p* value) of voxels with significantly increased (patient > controls; D, J, G, M) or decreased (patient < controls; E, K, H, N) BP_ND_ (*p* < 0.05; *p* ≤ 0.001 in red) in the cerebral hemispheres (D, E, J, K) and posterior fossa (G, H, M, N), together with the corresponding T2 MRI (C, F, I, L) for patients 3 and 11.

The decrease in white matter global BP_ND_ in patients with mitochondrial disease was mostly driven by 3 individuals (patients 3, 9, and 11) whose white matter BP_ND_ was severely reduced (see figure 1B). Patients 3 and 9 were respectively diagnosed with mitochondrial encephalomyopathy, lactic acidosis, and stroke-like episodes (MELAS) and maternally inherited diabetes and deafness (MIDD) caused by a heteroplasmic *MT-TL1* (m.3243A>G) mutation; patient 11 had mitochondrial neurogastrointestinal encephalopathy (MNGIE) caused by compound heterozygous *TYMP* mutations (table). The decrease in white matter BP_ND_ was most striking in the frontal and parietal lobes of the forebrain, where all 3 patients had relatively low radiotracer binding (figure 4A), and a large fraction of the tissue was composed of voxels with significantly decreased [^11^C]PK11195 BP_ND_ compared to controls (Figure 4, C–E and I–K). For patients 3 and 9 with *MT-TL1* mutations, BP_ND_ was also low in most other white matter ROIs (figure 4, A–H), but only patient 3 had a few small white matter lesions on MRI (table; figure 4C) and the MRI of patient 9 only showed generalized atrophy but no leukoencephalopathy. Patient 11 with *TYMP* mutations had widespread white matter abnormalities on MRI in the 4 lobes of both hemispheres and brainstem, compatible with MNGIE-related leukoencephalopathy (figure 4I and L). However, in contrast to the strong reductions in BP_ND_ in the frontal and parietal lobes (figure 4, A and K), radiotracer binding was normal or increased in the white matter of the temporal lobe and posterior fossa (figure 4, A, B, M, and N), as well as in deeper brain structures like pallidum and midbrain, and in the cerebellar white matter. Together, these highly variable binding patterns between brain regions and individuals indicate that different parts of the brain, and even different types of white matter, may respond in distinct ways to mitochondrial dysfunction, also when cells are affected by the same nuclear or mitochondrial gene mutation.

### Asymmetry of [^11^C]PK11195 Binding

**Figure 5:**
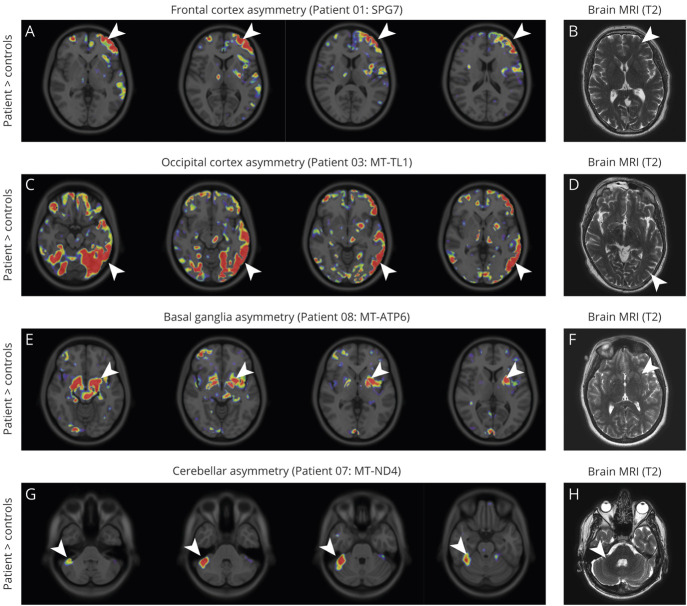
Asymmetry of [^11^C]PK11195 Binding (A–H) Overlay of −log_10_(*p* value) on 4 consecutive axial sections of the ICBM 152 2009a T1 MRI template for voxels with significantly increased (patient > controls) binding potential (*p* < 0.05; *p* ≤ 0.001 in red) (A, C, E, G) and the corresponding T2 MRI (B, D, F, H) for patients 1 (A and B), 3 (C and D), 8 (E and F), and 7 (G and H). Arrowheads indicate regions with strong asymmetry in binding potential.

Many of the [^11^C]PK11195 PET images showed asymmetric signal changes in specific regions of the brain (figure 5), without obvious corresponding abnormalities on the corresponding MRI. The most obvious examples of asymmetric radiotracer binding were in the cerebral cortex (frontal cortex in patient 1 with *SPG7* mutation; occipital cortex in patient 3 with an *MT-TL1* mutation; figure 5, A and C), the basal ganglia (patient 8, *MT-ATP6* mutation; figure 5E), or the cerebellum (patient 7 with an *MT-ND4* mutation; figure 5G). Asymmetric signal change was not clearly related to clinical presentation, as illustrated by the increased signal in the right lateral cerebellar hemisphere of a patient with LHON (patient 7; figure 5G) who was otherwise asymptomatic (table).

### Fraction of Voxels With [^11^C]PK11195 Binding Change Correlates With Clinical Severity

**Figure 6:**
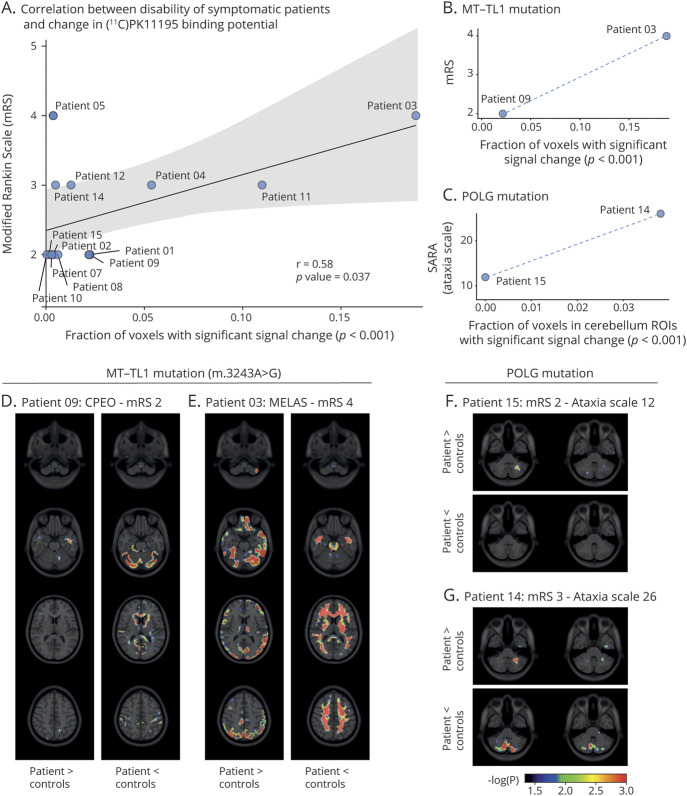
Clinical Severity Correlates With Changes in [^11^C]PK11195 Binding (A–C) Correlation between score on the (A, B) modified Rankin Scale (mRS) or (C) Scale for the Assessment and Rating of Ataxia (SARA) and the fraction of the brain (A and B) or cerebellum (C) where nondisplaceable binding potential (BP_ND_) was significantly (*p* < 0.001) different from the control population for all 13 symptomatic patients with mitochondrial disease (A; *r* 0.58; *p* value 0.037) or only for those patients with *MT-TL1* (B) or *POLG* (C) mutations. (D–G) Overlay of −log_10_(*p* value) for voxels with significantly increased (patient > controls) or decreased (patient < controls) [^11^C]PK11195 BP_ND_ (*p* < 0.05; *p* ≤ 0.001 in red) on the ICBM 152 2009a T1 MRI template at regular dorso-ventral intervals across the brain (D and E) or at the level of the cerebellum (F and G). ROI = region of interest.

We used the mRS to measure clinical disability in the 13 patients with symptomatic mitochondrial disease and the SARA^30^ to quantify the degree of ataxia. There was a significant positive correlation (*r* = 0.58; *p* = 0.037) between mRS and the fraction of voxels across all ROIs (gray and white matter) with a significant change (increase or decrease) in [^11^C]PK11195 binding (figure 6A): patients with a larger part of their brain affected by abnormal binding (either increased or decreased) had a higher chance of being more disabled as measured by the mRS. This was most striking within groups of patients with the same mutation (i.e., patients with the m.3243A>G *MT-TL1* mutation or the m.14487T>C *MT-ND6* mutation), where clinical symptoms as measured by mRS were always worse in those patients with a higher fraction of voxels with changed binding (figure 6, B, D, and E; figure e-3 available from Dryad: doi.org/10.5061/dryad.zs7h44j7s). For those patients with ataxia syndromes (i.e., 2 patients with a nuclear *POLG* mutation and 3 patients with a nuclear *SPG7* mutation), this trend was most striking for signal changes in the cerebellum, where a higher voxel fraction with aberrant [^11^C]PK11195 binding was associated with worse performance on the SARA scale (figure 6, C, F, and G; figure e-3 available from Dryad: doi.org/10.5061/dryad.zs7h44j7s). This demonstrates that, despite high interindividual variability in both radiotracer binding and clinical presentation, CNS binding of [^11^C]PK11195 is related to clinical severity.

## Discussion

We describe [^11^C]PK11195 PET brain imaging of the mitochondrial outer membrane protein TSPO in a group of representative patients with genetically confirmed mitochondrial disease. Our results demonstrate a correlation between the degree of abnormal radiotracer binding across the brain and clinical severity in symptomatic patients. In addition, we found that regional binding abnormalities may correspond to clinical presentation, are mutation-specific, and mostly occur in the absence of obvious structural changes on brain MRI.

[^11^C]PK11195 PET imaging of TSPO is widely used as a marker of neuroinflammation, due to its accumulation in activated microglia in the brain.^16-18^ Although this is probably valid for CNS disorders where the primary disease process is related to inflammation, TSPO abundance and radiotracer binding are likely to be differentially affected in many other noninflammatory conditions.^19^ In particular, a decrease in radiotracer binding, as previously observed in autism spectrum disorder,^20^ schizophrenia,^31^ or inactive multiple sclerosis lesions,^32^ is more difficult to reconcile with microglial activity or inflammation. Given its localization to mitochondria in all cell types of the CNS,^15^ it is conceivable that TSPO abundance in the brain is affected by pathologic changes associated with mitochondrial disease.

The 14 patients with mitochondrial disease had mutations in the mitochondrial or nuclear genomes causing a range of mitochondrial syndromes at various stages of their disease course (table), thus providing a heterogeneous, but representative case series. For 6 patients, the volume of abnormal [^11^C]PK11195 radiotracer binding across the brain exceeded the 95th percentile of the control population. In contrast to neuroinflammatory disorders, radiotracer binding was not simply increased, but patients and brain regions had variable patterns of reduced or enhanced BP_ND_. This indicates that mitochondrial disease is not necessarily accompanied by inflammation and microglial activity. These findings are in line with previous neuropathologic studies that only showed microglial proliferation in active stroke-like lesions in the temporal and occipital cortex of patients with MELAS,^33^ or late stages of Leigh syndrome lesions,^34^ but not elsewhere in the brain.^2,35,36^

Mitochondrial dysfunction frequently results in increased mitochondrial biogenesis and a redistribution of mitochondria within the cell. We found that, on average, in patients with mitochondrial disease, white matter was more likely to show reduced rather than increased TSPO abundance compared to controls. White matter is mostly composed of long-range axons and has relatively few cell bodies. This decrease in radiotracer binding in axon-rich regions of the brain is reminiscent of studies in cell culture^37^ and model organisms^38,39^ where genetic perturbation of mitochondrial function was associated with a reduction in mitochondrial density in the distal axons of neurons. In gray matter, where neuronal cell bodies are located, binding was more variable and frequently increased. Interestingly, in the soma of dopaminergic neurons of the substantia nigra, a gray matter region in the midbrain where we observed a significant increase in average BP_ND_ (figures 2B and 3C), elevated levels of mitochondrial protein expression have been found to coincide with respiratory chain deficiencies due to an m.8344A>G mutation or a single mtDNA deletion,^40^ and mtDNA copy number was shown to increase with aging.^41^ This suggests that increased radiotracer binding in gray matter regions of the brain could at least in part be explained by increased mitochondrial biogenesis with accumulation of mitochondria in the neuronal cell bodies. This is consistent with the “sick mitochondrion” hypothesis, in which preferential replication of defective mitochondria is thought to explain ragged-red fibers in skeletal muscle.^42-44^ Our findings indicate that the same mechanism may be at play in the brain. However, it is also possible that TSPO expression is independent of mitochondrial mass,^45^ and other cell types can also be affected, such as oligodendrocytes,^46^ or endothelium and smooth muscle cells of the cerebral vasculature.^2,36^ More extensive postmortem studies of mitochondrial mass and its relation to TSPO expression in the brain of patients with mitochondrial disease are required to fully support TSPO PET imaging as a novel noninvasive in vivo measure of mitochondrial density in the brain. Alternatively, it would be interesting to explore the validity of TSPO PET imaging of muscle, where neuropathologic correlates of mitochondrial biogenesis are more readily available.

CNS involvement in metabolic disorders such as mitochondrial disease is often thought to be bilateral and symmetric, with generalized brain or cerebellar atrophy, widespread white matter changes, or bilateral, symmetric basal ganglia lesions.^2,47^ Even lateralized parkinsonism symptoms are not necessarily accompanied by asymmetric lesions on brain MRI or dopamine transport (DAT-SPECT) imaging.^29^ Interestingly, many of our patients had large regions of asymmetric binding abnormalities, with otherwise normal or symmetric MRI. In the cerebellum of patients with cerebellar ataxia, we found significant reductions in [^11^C]PK11195 binding using regional BP_ND_ values corrected for atrophy with GTM partial volume correction. Although many patients had some degree of cerebellar atrophy, reduced binding was even observed in the absence of overt cerebellar atrophy as reported on MRI. Postmortem studies in patients with ataxia due to mitochondrial disease have found a reduction in mitochondrial density in the cerebellar cortex, possibly related to microscopic neuronal loss.^48^ Together, these findings suggest that changes in [^11^C]PK11195 binding may precede structural MRI abnormalities and could be more sensitive than MRI at detecting reduced neuronal or mitochondrial density.

This study used the prototypic TSPO PET tracer [^11^C]PK11195. Compared to higher-affinity second-generation TSPO PET tracers, such as [^11^C]PBR28 and [^11^C]DPA-713, [^11^C]PK11195 has lower transport into brain tissue and a lower ratio specific to nonspecific binding (except for low affinity binders), resulting in BP_ND_ estimates of inferior statistical quality.^49^ However, [^11^C]PK11195 has the advantage of obviating the need to divide the participants into binding affinity groups (high, mixed, low) driven by a polymorphism in the *TSPO* gene.^50^ In a small cohort, such as in this study, avoiding this subdivision is particularly advantageous. However, if much larger, multicenter studies employing TSPO PET imaging are conducted in the future on patients with mitochondrial disease, the cost, access to tracers or equipment, and reproducibility of imaging in different settings all will have to be taken into account, and the increased sensitivity offered by more accessible and cost-effective second-generation TSPO PET tracers labeled with fluorine-18 may be considered.

[^11^C]PK11195 BP_ND_ was quantified using reference tissue kinetic modeling, which requires a key additional assumption compared to gold standard kinetic modeling with an arterial input function; namely, that the reference tissue input is devoid of specific binding. Reference tissue inputs produced by the method employed in this study (supervised cluster analysis) were shown to have lower total distribution volumes than those obtained from the most commonly used anatomical reference tissue, the cerebellum.^27^ This supports the notion that reference tissue inputs from supervised cluster analysis better approximate the ideal situation of a null contribution from specific binding. To further improve the accuracy of the BP_ND_ estimates, the reference tissue model incorporates correction for vascular signal, including binding to endothelial TSPO receptors.^26^ Although reference tissue modeling of [^11^C]PK11195 PET data with a reference tissue input from supervised cluster analysis has been validated with arterial input function kinetic modeling,^27,51^ ideally this validation process should be extended to patients with mitochondrial disease.

Research into novel therapies of mitochondrial disease has been hampered by the lack of useful noninvasive biomarkers that measure disease severity and progression.^9,10^ Our findings suggest that PET imaging of TSPO has potential as a noninvasive biomarker of disease progression for CNS involvement in mitochondrial disease, given that binding abnormalities across the entire brain were significantly correlated with clinical severity. When we restricted the analysis to patients with the same genetic mutation, patients with more severe clinical presentations always had more extensive [^11^C]PK11195 binding abnormalities than their less disabled counterparts. Our cohort was too small and heterogeneous to demonstrate strong correlations between specific symptoms and regional binding characteristics. However, for patients with ataxia, symptom severity was clearly related to reduced radiotracer binding in the cerebellum. Larger natural history studies are needed that measure disease progression together with whole-brain and region-specific radiotracer binding, including in genetically homogeneous patient cohorts, to confirm the validity of TSPO PET as a reliable biomarker for interventional studies in thus far incurable mitochondrial diseases.

## Glossary

BP_ND_: nondisplaceable binding potential
FWHM: full width at half maximum
IQR: interquartile range
GTM: geometric transfer matrix
LHON: Leber hereditary optic neuropathy
MELAS: mitochondrial encephalomyopathy, lactic acidosis, and stroke-like episodes
MIDD: maternally inherited diabetes and deafness
MNGIE: mitochondrial neurogastrointestinal encephalopathy
mRS: modified Rankin Scale
PVC: partial volume correction
ROI: region of interest
SARA: Scale for the Assessment and Rating of Ataxia
TSPO: translocator protein
